# PD139 Investigation Of The Current Status Of New Health Technology Assessment In Korea And The Factors Influencing Assessment Results

**DOI:** 10.1017/S0266462324003751

**Published:** 2025-01-07

**Authors:** Jihyun Kang, Jinho Kim, Chae Min Shin, Boyoung Park

## Abstract

**Introduction:**

This study aimed to evaluate factors influencing assessment results in the new health technology assessment (nHTA) system in Korea.

**Methods:**

Publicly available HTA reports obtained from the nHTA website were selected as a data source. A total of 258 nHTA reports including 305 technologies were included in the analysis. The detailed information in the reports was classified into three major categories: technical characteristics, evaluation methods, and publication types. A chi-squared test was used to investigate differences in the levels of evidence (high, medium, or low) and assessment results (pass or fail) according to the three categories. Univariate and multivariate logistic regression analyses were performed to identify factors associated with the levels of evidence and assessment results.

**Results:**

nHTA reports that performed a meta-analysis and included randomized controlled trials for evidence synthesis were associated with higher levels of evidence. The corresponding odds ratios were 5.008 (95% confidence interval [CI]: 1.265, 18.826) and 27.052 (95% CI: 7.802, 103.330), respectively. The analysis showed that as the level of evidence increased, the likelihood of the assessment passing was significantly higher (odds ratio 2.789, 95% CI: 1.284, 6.057). However, univariate analysis indicated that performing a meta-analysis or including randomized controlled trials, both of which affect evidence level, did not have a statistically significant association with assessment results.

**Conclusions:**

This study is the first systematic analysis of the factors influencing the results of nHTA reports in Korea. While higher evidence levels were associated with positive assessment outcomes, factors affecting the evidence level itself did not directly influence assessment results. More efforts are needed to integrate high levels of evidence into assessments.

